# Are Direct-Acting Antivirals Effective and Safe for Hepatitis C Patients with Arterial Hypertension? Evidence from a Large Retrospective Real-World Study

**DOI:** 10.3390/v18070763

**Published:** 2026-07-12

**Authors:** Michał Brzdęk, Piotr Rzymski, Dorota Zarębska-Michaluk, Barbara Poniedziałek, Beata Lorenc, Hanna Berak, Włodzimierz Mazur, Justyna Janocha-Litwin, Magdalena Tudrujek-Zdunek, Marek Sitko, Jakub Klapaczyński, Robert Flisiak

**Affiliations:** 1 Collegium Medicum, Jan Kochanowski University, 25-317 Kielce, Poland; michal.brzdek@gmail.com; 2 Department of Environmental Medicine, Poznan University of Medical Sciences, 60-806 Poznań, Poland; bpon@ump.edu.pl; 3 Department of Infectious Diseases and Allergology, Jan Kochanowski University, 25-317 Kielce, Poland; dorota1010@tlen.pl; 4 Pomeranian Center of Infectious Diseases, Medical University, 80-214 Gdańsk, Poland; lormar@gumed.edu.pl; 5 Outpatient Clinic, Hospital for Infectious Diseases in Warsaw, 01-201 Warsaw, Poland; hberak@zakazny.pl; 6 Clinical Department of Infectious Diseases in Chorzów, Medical University of Silesia, 40-055 Katowice, Poland; wlodek.maz@gmail.com; 7 Department of Infectious Diseases and Hepatology, Wrocław Medical University, 50-367 Wrocław, Poland; justynajanocha@o2.pl; 8 Department of Infectious Diseases, Medical University of Lublin, 20-059 Lublin, Poland; magdalena.tudrujek@gmail.com; 9 Department of Infectious and Tropical Diseases, Jagiellonian University, 31-088 Kraków, Poland; sitkomar@o2.pl; 10 Department of Internal Medicine and Hepatology, The National Institute of Medicine of the Ministry of Interior and Administration, 02-507 Warszawa, Poland; klapaj@gmail.com; 11 Department of Infectious Diseases and Hepatology, Medical University of Białystok, 15-089 Białystok, Poland

**Keywords:** hypertension, hepatitis C virus, direct-acting antivirals, treatment effectiveness, treatment safety, sustained viral response

## Abstract

**Background/Objectives**: Arterial hypertension (AH) and hepatitis C virus (HCV) infection are interlinked, with AH increasing the risk of severe liver disease and HCV contributing to cardiovascular issues. Treating HCV in hypertensive patients is critical, though data on direct-acting antivirals (DAAs) in this group remain limited. **Methods**: This retrospective study evaluated the effects of DAAs in AH patients with HCV using data from the 2015–2023 EpiTer-2 project, a multicenter study in Poland. **Results**: Among the 18,968 HCV-infected DAA-treated patients, 5976 had AH. These patients were older, predominantly women, and had higher rates of obesity, comorbidities, cirrhosis, hepatocellular carcinoma, and genotype 1b infection. Sustained virologic response rates were high and comparable between the AH and non-AH groups in the intent-to-treat (94.8% vs. 94.2%) and per-protocol analyses (97.6% vs. 97.6%). AH was not independently associated with treatment failure (OR 0.87, 95% CI: 0.69–1.10). Predictors of failure included genotype 3, decompensated liver function, cirrhosis, thrombocytopenia, and treatment with asunaprevir + daclatasvir. While therapy discontinuation was more common in the AH patients, most completed treatment, with fatigue being the most frequent adverse event. Although not directly evaluated, the overall safety outcomes suggest that potential drug–drug interactions with antihypertensive therapies are unlikely to have a major clinical impact in routine practice. **Conclusions**: This study highlights the safety and efficacy of DAAs in AH patients and emphasizes the importance of early HCV detection and treatment in this population.

## 1. Introduction

Hepatitis C virus (HCV) infection is a significant global health challenge, affecting an estimated 50 million people worldwide and accounting for about 1.0 million new infections per year [1]. Despite years of effort and various approaches, HCV infection remains unpreventable by a vaccine [2]. Spontaneous clearance is a relatively rare event, and the majority of infected individuals will progress to chronic hepatitis, which is a leading cause of liver-related morbidity and mortality, contributing to the development of cirrhosis, hepatocellular carcinoma (HCC), and liver failure [3,4]. Beyond its direct impact on hepatic function, HCV infection has been linked to several extrahepatic manifestations, including metabolic disorders, immunological phenomena, endocrinological abnormalities, and cardiovascular diseases, highlighting its systemic nature [5,6].

One of the most critical comorbid conditions affecting patients with chronic HCV is arterial hypertension (AH) [7], which itself is a global public health burden, affecting an estimated 1.28 billion individuals aged 30–79 years. In adults, it is defined as a systolic blood pressure (SBP) of 140 mmHg or higher and/or a diastolic blood pressure (DBP) of 90 mmHg or higher, and remains a major modifiable factor related to morbidity and mortality in middle- and high-income countries [8]. Common AH risk factors include advanced age, obesity, lack of physical activity, high salt intake, excessive alcohol consumption, stress, and a family history of hypertension [9]. Although its global prevalence is projected to decrease in the upcoming decades, it will remain a significant health concern in many regions [10].

The prevalence of AH is notably higher in patients with chronic HCV infection compared to that in the general population, suggesting a potential interaction between viral pathophysiology and cardiovascular risk [11]. HCV-induced chronic inflammation, oxidative stress, and metabolic disturbances, such as insulin resistance, may contribute to the development and worsening of hypertension in affected individuals [7,12]. The mechanisms underlying this association are complex and involve several pathways of virus–host interaction. Chronic HCV infection is characterized by persistent systemic inflammation, with increased production of pro-inflammatory cytokines, including interleukin-6 and tumor necrosis factor-α, as well as elevated levels of C-reactive protein and fibrinogen, all of which may contribute to vascular injury, increased vascular tone, and the development of hypertension [13,14]. In addition, HCV has been associated with endothelial dysfunction and increased arterial stiffness, resulting in impaired vasodilation and adverse vascular remodeling [15,16]. As liver fibrosis progresses, several neurohormonal mechanisms may further promote blood pressure elevation, including the activation of the sympathetic nervous system, increased endothelin-1 production, the stimulation of the renin–angiotensin–aldosterone system (RAAS), sodium retention, and vasopressin-mediated fluid imbalance [7]. However, the relationship between HCV and blood pressure is not uniform across all stages of liver disease. While chronic infection and compensated liver disease are frequently associated with higher blood pressure, patients with advanced cirrhosis may become normotensive or hypotensive due to cirrhotic cardiomyopathy and hyperdynamic circulation, which reduce systemic vascular resistance [17]. Furthermore, hypertension exacerbates the risk of severe liver disease and cardiovascular complications in patients with HCV, creating a bidirectional relationship that complicates the management of both conditions [18,19]. Overall, AH can increase the complexity of clinical management due to potential drug–drug interactions, coexisting renal impairment, an elevated cardiovascular risk, and its frequent overlap with advanced liver disease.

In the last decade, the advent of direct-acting antivirals (DAAs) has revolutionized the treatment landscape of HCV [20]. These agents, which target specific steps of the viral replication cycle, have shown remarkable efficacy in achieving sustained virologic response (SVR) with high cure rates across patient groups, including those with comorbidities [21,22,23,24]. However, the effects of HCV treatment in patients with AH remain incompletely understood. Limited evidence suggests that successful HCV treatment with DAAs may improve metabolic profiles, vascular health, and inflammation, positively influencing blood pressure control [25,26].

Therefore, it is essential to comprehensively analyze the effectiveness and safety of DAA treatment in HCV-infected patients with AH. Such analyses are particularly needed in countries with a relatively high burden of both diseases, such as Poland, where the number of patients registered with AH was nearly 11 million in 2022, with a prevalence in adults of 35%, while 0.5% of the population was estimated to be infected [27]. Therefore, this study aimed to assess the effectiveness and safety of HCV treatment with DAAs in patients with AH in comparison to those without this comorbidity, and to identify clinical factors associated with treatment outcomes in this population, through a retrospective, real-world analysis of the Polish adult population of HCV-infected patients treated with DAAs between 2015 and 2023.

## 2. Materials and Methods

### 2.1. Studied Population and Data Collection

Data of 18,968 patients treated with DAAs for chronic hepatitis C (CHC) between 1 July 2015, and 31 December 2023, from the EpiTer-2 observational study, the database of CHC treatment in Poland run by the Polish Association of Epidemiologists and Infectiologists, one of the largest in Europe, was analyzed. It includes information from 22 national hepatology centers and reflects real-world treatment experiences. The choice of CHC therapy was at the physician’s discretion, following the Polish Group of Experts guidelines for HCV and the National Health Fund in Poland, with concomitant medications routinely reviewed prior to treatment initiation to minimize potential drug–drug interactions. Data were collected retrospectively and covered variables such as age, sex, body mass index (BMI), liver disease severity, HCV genotype (GT), viral load, HIV and/or HBV coinfection, prior and current CHC treatments, the presence of hepatocellular carcinoma, HCV viral load, alanine transaminase (ALT) activity, albumin, hemoglobin, creatinine, and platelet levels. HBV coinfection was identified based on HBs antigen positivity. Abnormal values of biochemical parameters were considered as follows: ALT > 35 IU/I, albumin < 3.5 g/dL, bilirubin > 1.2 mg/dL, hemoglobin (women: <12 g/dL, men: <13 g/dL), creatinine > 1.2 mg/dL, and platelet count < 140 × 1000/µL.

The study was approved by the Bioethics Committee of Jan Kochanowski University in Kielce by resolution No. 57/2024 on 25 July 2024. 

### 2.2. Arterial Hypertension Diagnosis

AH was diagnosed based on a detailed medical interview and the analysis of medical documentation. When patients could not provide complete information, additional data were obtained from family members. The analysis of medical records included previous physician-diagnosed arterial hypertension and was based on the European Society of Cardiology clinical guidelines that were in force at the time of diagnosis. Only records with clinically confirmed hypertension were included in the study. Information on antihypertensive pharmacotherapy, blood pressure control, and hypertension staging was not systematically collected in the database and, therefore, was not included in the analysis.

### 2.3. Liver Disease Severity Evaluation

The severity of liver disease was evaluated using non-invasive fibrosis assessment methods, including transient elastography with the use of FibroScan (Echosens, Paris, France) or shear-wave elastography with the Aixplorer device (SuperSonic Imagine, Aix-en-Provence, France). Following the METAVIR scoring system and the European Association for the Study of the Liver guidelines, a threshold of 13 kPa was used to identify individuals likely to have cirrhosis (F4) [28]. These patients were further evaluated for liver function decompensation (both in the past and at the initiation of antiviral therapy) and assessed using the Child-Pugh (CP) scale. Patients with a CP score of B or C were classified as decompensated. Additionally, data were gathered on a history of HCC diagnosis.

### 2.4. Treatment Effectiveness Evaluation

Antiviral therapy was deemed successful if the HCV RNA was undetectable in the serum at least 12 weeks after treatment completion, indicating that the patient had achieved a sustained virologic response (SVR). Patients with a detectable viral load were classified as virologic failures, while those lost to follow-up without HCV RNA testing at the 12-week mark were categorized as non-virologic failures and excluded from the per-protocol (PP) analysis.

### 2.5. Treatment Safety Evaluation

Safety outcomes were recorded during the treatment process and monitored for 12 weeks after completion. During the treatment and follow-up periods, data were gathered on any alterations to or cessation of the treatment regimen, occurrences of adverse events (AEs), severe adverse events, and any fatalities, along with evaluations of their association with the antiviral therapy. Adverse events explicitly associated with liver function, such as gastrointestinal bleeding, ascites, and encephalopathy, were carefully observed in patients with cirrhosis.

### 2.6. Statistical Analyses

Statistical analyses were performed using Statistica v. 13 (StatSoft, Tulsa, OK, USA) and MedCalc v. 15.8 (MedCalc Software Ltd., Ostend, Belgium). The entire study population was included in the intent-to-treat (ITT) analysis, whereas the PP analysis included patients who underwent HCV RNA testing 12 weeks after completing treatment. Differences in event frequencies between groups with and without hypertension were assessed using Pearson’s χ^2^ test or Fisher’s exact test (when the number of observations was <10). The differences in data expressed on the interval scale were evaluated with the t-test. Based on the results of univariate analyses comparing hypertensive and non-hypertensive patients and comparing hypertensive patients who achieved and did not achieve SVR, logistic multiple regression models were used to predict the odds of no response to HCV treatment. Potential multicollinearity among candidate variables was assessed during multivariable model construction. Variables representing overlapping clinical constructs were not entered simultaneously into the final model, and no evidence of clinically relevant multicollinearity was identified among the retained predictors. A *p*-value < 0.05 was considered statistically significant.

## 3. Results

### 3.1. General Characteristics of Patients

Of the 18,968 patients treated for HCV infection, 5976 (31.5%) were diagnosed with AH. They were significantly older, predominantly female, and had a higher prevalence of obesity, coronary artery disease, diabetes, renal disease, malignancies other than HCC, decompensation of liver function, and cirrhosis. Additionally, they more frequently had a history of HCC, were more often infected with GT1b, but less frequently with GT1a, GT3, and GT4, and were less frequently coinfected with HIV and HBV. In addition, hypertensive patients more frequently had albumin, bilirubin, creatinine, hemoglobin, and platelet concentrations outside the normal range (Table 1). The viral load did not differ between the hypertensive and non-hypertensive groups (mean ± SD HCV RNA 2.52 ± 0.77 vs. 2.33 ± 0.69 × 106 IU/mL, *p* = 0.08).

Most patients with and without AH were treatment-naïve. However, the former group had a 3.8% higher frequency of a history of previous treatment failure (Table 2), with 91.4% of them having been unsuccessfully treated with IFN-based therapy. Discontinuation of the previous therapy due to adverse events was also more common in this group (Table 2). In the current treatment, these patients received genotype-specific and RBV-containing regimens more frequently than those treated with DAAs (Table 2).

### 3.2. Treatment Effectiveness

**Figure 1:**
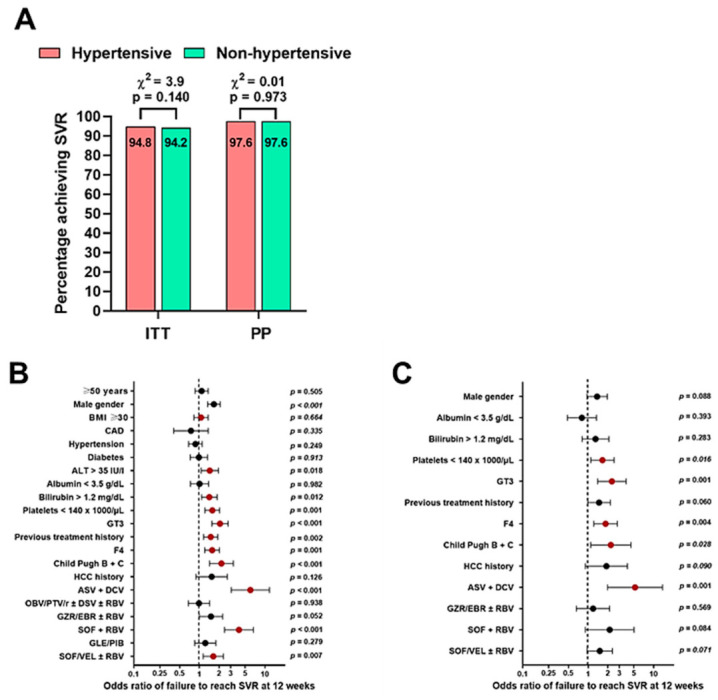
(**A**)The sustained virological response rate (SVR) in the group with hypertension (n = 5976) compared to the group without hypertension (n = 12,992) in intention-to-treat and per protocol analyses. (**B**) Logistic multiple regression results (OR and 95%CI) on the association between lack of SVR, hypertension, and parameters that differentiated hypertensive patients in univariate analysis. Red dots for OR highlight significant results. (**C**) Logistic multiple regression results (OR and 95%CI) on the association between lack of SVR in hypertensive patients and their characteristics that differed from hypertensive patients achieving SVR. Red dots for OR highlight significant results.

The effectiveness of DAA therapy was very high in the hypertensive group, with SVR at 12 weeks after treatment completion reaching 94.8% and 97.6% in the ITT and PP analyses, respectively. Treatment effectiveness in these patients did not differ from that in patients without hypertension (Figure 1A). In the univariate analysis of the entire group of 18,968 patients, SVR was not achieved more frequently by men, individuals with decreased albumin and increased bilirubin, thrombocytopenia, abnormal liver function (defined as B and C scores in the CP scale), cirrhosis, HCC history, GT3 infection, history of previous treatment failure, and those treated with ASC + DCV, SOF + RBV, SOF/VEL ± RBV, and RBV-containing regimens (Table 3). These variables plus hypertension were selected for the multivariate logistic regression model, revealing that male gender, GT3 infection, previous treatment history, treatment with ASC + DCV, SOF + RBV, SOF/VEL ± RBV, CP score B or C, cirrhosis, ALT level > 35 IU/I, bilirubin concentration > 1.2 mg/dL and thrombocytopenia were independent factors predicting failure to achieve SVR in the studied group (Figure 1B). Hypertension was not a predictor of treatment failure.

Individuals with AH who did not achieve SVR did not differ in age, BMI, or the prevalence of comorbidities (autoimmune diseases, malignancies other than HCC, coronary artery disease, diabetes, and renal disease), but more frequently men, had B/C Child-Pugh Score, liver cirrhosis, HCC history, and concentration of albumin, bilirubin and platelets outside the reference range, and were less commonly infected with GT1b but more often with GT3 (Table 3). In multivariate analysis, SVR failure was associated only with GT3 infection, decompensated liver function, cirrhosis, thrombocytopenia, and ASC + DCV treatment regimen (Figure 1C).

### 3.3. Treatment Safety

Patients with AH were more often lost to follow-up than non-hypertensive individuals (3.5% vs 2.2%). Although the vast majority of them did not report any AEs, they were more prone to therapy discontinuation, particulary due to AEs, which were more frequent in this group, including serious AEs (Table 4). Hypertensive patients more commonly experienced fatigue, anemia, and itchy skin, with approximately one-fifth reporting at least one AE. Mortality was significantly higher in the group of patients with AH, with a total of 47 deaths, of which seven were associated with the advancement of liver disease. In the group without hypertension, 68 deaths were reported, with 15 linked to the advancement of liver disease. None of the deaths were considered by the treating physician to be associated with antiviral treatment.

## 4. Discussion

The present work provides a broad overview of the characteristics of HCV-infected patients with AH using data from an extensive national multicenter database. To the best of our knowledge, this is the first study to specifically address the problem of treating chronic hepatitis C with various DAA regimens in such a group. It encompasses a 9-year period of interferon-free therapies based on DAAs, allowing for a comprehensive assessment of their real-world effectiveness and safety in this specific group of patients, who constitute approximately one-third of all individuals treated for hepatitis C in Poland. As documented previously, hypertension is a significant comorbidity in HCV-infected patients. Considering that hypertension will continue to cause a substantial health burden in the subsequent decades, the findings of our study are relevant for the future management of hepatitis C. Because the study covered the entire period since the introduction of DAAs, it included patients treated with both earlier and currently recommended antiviral regimens, thereby reflecting real-world clinical practice and the evolution of HCV treatment over time.

As shown, the hypertensive patients tended to be older, more frequently female, and had a higher prevalence of obesity. This observation is generally in line with the typical characteristics of individuals with AH in Poland, who are most often over 60 years old, are predominantly women, and have an increased BMI, with at least one-third having obesity [27,29]. On the other hand, it is worrisome that hypertensive patients in our cohort tended to be treated with DAAs for HCV infection at an older age. It indicates that treatment in these patients was potentially introduced after a prolonged HCV infection, having a more adverse effect on their liver health. Such an assumption is further confirmed by the increased prevalence of liver function impairment, cirrhosis, and a history of HCC in these individuals. Although DAA treatment can eliminate the virus and cease its further action, it does not reverse the significant damage already done to hepatic function [30,31]. However, one should also note that these patients had a slightly higher frequency of a history of previous treatment failure and its discontinuation due to AEs, almost exclusively after IFN-based therapies. This phenomenon could also influence the use of DAAs in hypertensive individuals at an older age and with more advanced liver disease. IFN-based therapies were characterized by a wide array of debilitating side effects, including hematological and neurological ones, making the therapy challenging to tolerate for many patients, in addition to having a long duration, requiring subcutaneous injections, and having moderate effectiveness [32,33,34,35]. The registration of DAAs, followed by the introduction of pangenotypic regimens, has revolutionized the treatment in many different populations, and, as shown in our study, also in individuals with AH. Nevertheless, it could be argued that patients with AH and those in a prehypertensive state should be targeted for screening of HCV infection to avoid late detection. Such screening could be achieved using classical laboratory methods and rapid cassette anti-HCV antibody tests, which are inexpensive, have a generally recognized diagnostic value, and have an execution time comparable to measuring blood pressure in a doctor’s office [36].

The detection of HCV infection as soon as possible is crucial, as our study unequivocally shows that it can be successfully treated in patients with AH with high rates of achieved SVR, not differing from those observed in patients without this comorbidity. We also demonstrate that hypertension is not an independent predictor of treatment failure. The identified independent negative predictors of not achieving SVR included GT3 infection, the decompensation of hepatic function, and the presence of liver cirrhosis, which is in line with observations from treating hepatitis C in the general population. Despite the significant revolution in HCV therapy and the opportunity to achieve SVR in the vast majority of patients, GT3 infections remain challenging to treat due to their association with a higher rate of steatosis, accelerated liver fibrosis, and high frequencies of resistance-associated substitutions to nonstructural protein 5A inhibitors [37,38,39].

In addition, thrombocytopenia and treatment with ASV + DCV were also found to predict failure to reach SVR in hypertensive patients. The former is associated with hepatocellular damage and hepatic fibrosis [40,41,42]. This is an important observation also in the context of a higher frequency of a lower platelet count in AH patients compared to other individuals treated with DAAs, and because some medications to treat increased blood pressure, especially calcium channel blockers, may induce thrombocytopenia [43,44,45]. In turn, the ASV + DCV regimen was registered only for treating patients infected with GT1b and eventually considered suboptimal following a meta-analysis of nine clinical trials that yielded a cumulative efficacy of 90% in the treatment-naïve group and 83% in non-responders to IFN-based therapy [46]. Importantly, though, no pangenotypic regimen was shown in the present study to be related to treatment failure.

The present study also documents that DAA treatment in hypertensive patients had a good safety profile, with the vast majority of patients completing it and not reporting any AEs. Nevertheless, the frequency of therapy discontinuation due to AEs, the occurrence of at least one AE, and the onset of serious AEs were more common in this group compared to individuals without arterial hypertension. This observation may have several explanations. Firstly, hypertensive patients tended to be older, while older age is associated with a diminished ability to metabolize different pharmaceuticals, increasing the risk of side effects. Secondly, the more advanced liver disease observed in hypertensive patients infected with HCV may reduce the hepatic capacity to process medications [47]. Thirdly, comorbid conditions, such as obesity, diabetes, kidney disease, and cardiovascular disease, were more frequent in patients with AH, making them more vulnerable to drug-induced complications and drug–drug interactions (DDIs) [48].

Our observations demonstrating a generally favorable safety profile of DAA therapy in hypertensive patients are particularly important in the context of previous reports suggesting that DDIs between DAAs and antihypertensive agents may represent an underappreciated clinical issue. Some real-world analyses indicate that antihypertensive drugs, particularly calcium channel blockers, diuretics, and agents metabolized via cytochrome P450 pathways, are among the most frequently involved in clinically significant DDIs during DAA therapy [49,50,51,52,53,54]. Mechanistically, several DAAs, especially ritonavir-boosted regimens, inhibit CYP3A4 and transport proteins such as P-glycoprotein, leading to increased systemic exposure to commonly used antihypertensives, including amlodipine and other calcium channel blockers, thereby potentiating their pharmacodynamic effects [52].

A large Taiwanese multicenter cohort demonstrated that patients with documented DAA–antihypertensive DDIs experienced significantly greater reductions in blood pressure and an increased incidence of hypotensive episodes; notably, among patients treated with glecaprevir/pibrentasvir and presenting a moderate comorbidity burden, the odds of hypotension were approximately 4.5-fold higher in the presence of DDIs [55]. Similarly, observational data from U.S. clinical practice indicate that coadministration of ritonavir-boosted DAA regimens frequently necessitated dose reductions in antihypertensive drugs or intensified blood pressure monitoring due to the risk of excessive drug exposure and symptomatic hypotension [52]. Territory-wide analyses further confirm that calcium channel blockers constitute one of the most common classes requiring monitoring or adjustment during DAA therapy [51].

However, our findings suggest that, despite the theoretical and previously reported risks, these interactions do not appear to translate into a substantial clinical burden when DAA therapy is administered in routine practice under appropriate medical supervision. In our cohort, the vast majority of hypertensive patients completed therapy without adverse events, and although AEs, including serious ones and treatment discontinuations, were more frequent than in non-hypertensive individuals, their overall incidence remained low. This indicates that potential DDIs are likely being effectively mitigated through appropriate regimen selection, dose adjustments, and clinical monitoring, rather than constituting a major barrier to safe and effective antiviral treatment.

Importantly, current expert consensus does not recommend discontinuation or empirical down-titration of essential antihypertensive therapy solely to accommodate antiviral treatment. Instead, a more rational strategy involves selecting DAA regimens with a lower interaction potential (most notably sofosbuvir-based combinations) while implementing appropriate dose adjustments and close clinical monitoring, particularly during the initial weeks of therapy [49,53,54,56]. Our real-world data indirectly support this approach, demonstrating that high treatment completion rates and favorable safety outcomes can be achieved without compromising the management of coexisting hypertension. This individualized approach is especially critical in patients with coexisting renal impairment, advanced liver disease, or high cardiovascular risk, where both undertreatment and overtreatment of hypertension may have serious consequences.

At the same time, the addition of HCV treatment may exacerbate baseline symptoms such as fatigue, particularly in hypertensive individuals with obesity and diabetes who may already experience reduced energy levels and endurance [57,58]. While DDI-related hemodynamic changes, including transient hypotension, may contribute to such symptoms, our data suggest that their overall clinical impact is limited and manageable in most patients. Therefore, careful but not overly restrictive management of potential DDIs appears sufficient to maintain both the safety and effectiveness of DAA therapy in this population.

The study’s strengths include a large sample size that increases the statistical power of the analysis, improving the reliability and generalizability of the results. Moreover, the study provides a real-world perspective, enhancing the external validity of the findings, as they reflect clinical outcomes in everyday healthcare settings, unlike tightly controlled clinical trials. Furthermore, the study provides a detailed characterization of patients, including demographic data, comorbidities, viral genotype, and treatment regimens, enabling more nuanced subgroup analyses and identification of independent predictors of treatment failure in the entire cohort and, specifically, in hypertensive patients. Last but not least, the analysis spans a 9-year period (2015–2023), which allows for the assessment of long-term outcomes and trends in DAA treatment effectiveness and safety among hypertensive patients.

The study limitations should also be stressed. The retrospective nature of the analysis makes it more prone to data entry errors, incomplete data, possible physician bias, and the underreporting of AEs. Moreover, detailed information on antihypertensive treatment regimens, blood pressure control, and metabolic liver comorbidities was not consistently available; therefore these aspects were beyond the scope of the present analysis. Furthermore, the present study was not designed to assess the effect of HCV eradication on blood pressure control or the course of hypertension-related comorbidities. Such analyses would require prospective longitudinal follow-up. Therefore, our findings should only be interpreted as evidence of the effectiveness and safety of DAA therapy in patients with AH. Additionally, information on hypercholesterolemia and lipid-lowering therapy, including statin use, was not consistently available in the database. This represents a limitation because statins are commonly prescribed in patients with arterial hypertension [59]. Nevertheless, the overall favorable safety profile, low rate of treatment discontinuation, and high treatment completion observed in our cohort suggest that potential statin-related drug–drug interactions and other drug–drug interactions did not result in a substantial clinical burden in routine practice, although this issue warrants further investigation in studies with more detailed pharmacotherapy data. Since it was a real-world analysis, no strict rigor during therapy could be imposed, unlike in clinical trials.

## 5. Conclusions

The present study shows that the treatment of chronic hepatitis C in patients with AH is highly effective and safe. While hypertension itself is not a predictor of treatment failure, other factors, such as GT3 infection, the presence of decompensated liver disease, cirrhosis, and thrombocytopenia, significantly affect outcomes in hypertensive patients. In addition, the findings underscore the importance of early detection and timely treatment of HCV infection in patients with AH, who often present at an older age with more advanced liver disease. Although DDIs were not directly assessed, the favorable safety profile observed suggests that potential interactions between DAAs and antihypertensive medications are unlikely to translate into clinically significant adverse outcomes when therapy is appropriately managed. These results reinforce the need for targeted screening strategies, particularly in hypertensive individuals, to optimize treatment success and reduce the long-term burden of HCV in this population. This retrospective real-world study used a single observational approach to evaluate the effectiveness and safety of antiviral therapy; therefore, the findings should be confirmed and complemented by further studies.

## Figures and Tables

**Table 1:** Characteristics of patients with and without arterial hypertension treated for chronic hepatitis C with direct-acting antivirals.

Parameter	Hypertensive Patients (n = 5976)	Non-Hypertensive Patients (n = 12,992)	*p*-Value
**Age, mean ± SD**	61.2 ± 12.1	46.5 ± 13.3	<0.0001
**Age ≥ 50, % (n)**	82.9 (4952)	38.6 (5017)	<0.0001 (*χ*^2^ = 3214.1)
**Men/Women, % (n)**	46.2/53.8 (2758/3218)	52.1/47.9 (6762/6230)	<0.0001 (*χ*^2^ = 56.9)
**BMI, mean ± SD**	27.7 ± 4.8	25.5 ± 4.2	<0.0001
**BMI ≥ 30, % (n)**	27.1 (1581)	13.2 (1677)	<0.0001 (*χ*^2^ = 541.7)
**Comorbidities**			
**Autoimmune diseases, % (n)**	2.3 (135)	1.9 (251)	0.138 (*χ*^2^ = 2.2)
**Malignancies other than HCC, % (n)**	2.9 (171)	1.8 (237)	<0.0001 (*χ*^2^ = 20.9)
**Diabetes, % (n)**	24.7 (1473)	5.4 (706)	<0.0001 (*χ*^2^ = 1486.2)
**Renal disease, % (n)**	7.6 (456)	1.8 (234)	<0.0001 (*χ*^2^ = 396.8)
**CAD, % (n)**	8.6 (514)	1.2 (154)	<0.0001 (*χ*^2^ = 662.5)
**ALT > 35 IU/I, % (n)**	79.2 (4734)	79.0 (10,263)	0.727 (*χ*^2^ = 0.1)
**Albumin < 3.5 g/dL, % (n)**	15.6 (931)	10.0 (1296)	<0.0001 (*χ*^2^ = 124.0)
**Bilirubin > 1.2 mg/dL, % (n)**	13.4 (802)	12.1 (1575)	0.01 (*χ*^2^ = 6.2)
**Hemoglobin** **(women/men, <12/13 g/dL), % (n)**	11.9 (709)	9.9 (1284)	0.004 (*χ*^2^ = 17.1)
**Platelets < 140 *×* 1000/µL, % (n)**	29.4 (1759)	20.0 (2603)	<0.0001 (*χ*^2^ = 204.2)
**Creatinine > 1.2 mg/dL, % (n)**	8.4 (500)	2.9 (373)	<0.0001 (*χ*^2^ = 281.6)
**Liver fibrosis**			
**F0, % (n)**	1.3 (76)	3.5 (447)	<0.0001 (*χ*^2^ = 71.8)
**F1, % (n)**	27.4 (1608)	45.3 (5780)	<0.0001 (*χ*^2^ = 532.1)
**F2, % (n)**	18.4 (1082)	19.3 (2465)	0.155 (*χ*^2^ = 2.0)
**F3, % (n)**	17.5 (1026)	12.0 (1528)	<0.0001 (*χ*^2^ = 75.1)
**F4, % (n)**	35.5 (2088)	19.9 (2532)	<0.0001(*χ*^2^ = 530.4)
**Child-Pugh Score**			
**B, % (n)**	3.7 (216)	3.0 (385)	0.02 (*χ*^2^ = 5.7)
**C, % (n)**	0.1 (8)	0.2 (28)	0.283
**HCC history, % (n)**	2.4 (145)	1.2 (153)	<0.0001 (*χ*^2^ = 41.3)
**HCV genotype**			
**1a, % (n)**	1.7 (101)	5.8 (759)	<0.0001 (*χ*^2^ = 163.0)
**1b, % (n)**	81.2 (4855)	70.3 (9133)	<0.0001 (*χ*^2^ = 253.2)
**1, % (n)**	2.3 (138)	1.9 (242)	0.130 (*χ*^2^ = 2.3)
**2, % (n)**	0.2 (9)	0.3 (44)	0.03
**3, % (n)**	9.3 (557)	13.8 (1788)	<0.0001 (*χ*^2^ = 74.5)
**4, % (n)**	3.6 (217)	5.4 (700)	<0.0001 (*χ*^2^ = 27.5)
**5, % (n)**	0.0 (0)	0.02 (1)	-
**6, % (n)**	0.03 (2)	0.03 (4)	1.0
**Unknown, % (n)**	1.6 (96)	2.5 (320)	<0.0002 (*χ*^2^ = 14.0)
**HBV coinfection, % (n)**	0.8 (46)	1.2 (144)	0.03 (*χ*^2^ = 4.7)
**HIV coinfection, % (n)**	2.1 (120)	7.9 (1016)	<0.0001 (*χ*^2^ = 244.8)

ALT: alanine transaminase; CAD: Coronary artery disease; BMI: Body mass index; F: fibrosis; HBV: Hepatitis B virus; HCC: hepatocellular carcinoma; HCV: hepatitis C virus; HIV: Human immunodeficiency virus.

**Table 2:** Characteristics of treatment of chronic hepatitis C patients with and without arterial hypertension.

Parameter	Hypertensive Patients (n = 5976)	Non-Hypertensive Patients (n = 12,992)	*p*-Value
**Previous treatment history**			
**Treatment naïve, % (n)**	77.5 (4634)	82.3 (10,694)	<0.0001 (*χ*^2^ = 60.0)
**History of treatment failure, % (n)**	19.5 (1167)	15.7 (2045)	<0.0001 (*χ*^2^ = 41.7)
**Discontinuation of therapy due to AEs, % (n)**	2.9 (175)	1.9 (250)	<0.0001 (*χ*^2^ = 134.8)
**Current treatment**			
**ASV + DCV** **, % (n)**	1.0 (62)	0.6 (73)	0.0003 (*χ*^2^ = 13.1)
**SOF/LDV ± RBV** **, % (n)**	19.2 (1145)	14.8 (1916)	<0.0001 (*χ*^2^ = 58.9)
**OBV/PTV/r ± DSV ± RBV** **, % (n)**	21.8 (1300)	21.3 (2764)	0.455 (*χ*^2^ = 0.6)
**GZR/EBR ± RBV** **, % (n)**	16.6 (991)	12.4 (1610)	<0.0001 *(χ*^2^ = 60.8)
**SOF + SMV ± RBV,** **% (n)**	0.1 (3)	0.1 (7)	1.0
**SOF + RBV,** % (n) *	1.6 (98)	1.9 (246)	0.224 (*χ*^2^ = 1.5)
**SOF + DCV ± RBV**	0.2 (15)	0.2 (31)	0.872 (*χ*^2^ = 0.02)
**GLE/PIB** **, % (n)**	18.5 (1107)	29.5 (3827)	<0.0001 (*χ*^2^ = 254.2)
**GLE/PIB + SOF + RBV** **, % (n)**	0.03 (2)	0.04 (5)	1.0
**SOF/VEL ± RBV** **, % (n)**	20.6 (1232)	18.8 (2445)	0.004 (*χ*^2^ = 8.4)
**SOF/VEL/VOX, % (n)**	0.4 (21)	0.5 (68)	0.107 (*χ*^2^ = 2.6)
**Current-RBV-containing regimens, % (n)**	17.6 (1053)	12.4 (1616)	<0.0001 (*χ*^2^ = 90.9)

AE: adverse effects; ASV: Asunaprevir; DCV: Daclatasvir; DSV: Dasabuvir; EBR: Elbasvir; GLE: Glecaprevir; GZR: Grazoprevir; LDV: Ledipasvir; OBV: Ombitasvir; r: Ritonavir; PTV: Paritaprevir; PIB: Pibrentasvir; RBV: Ribavirin; SMV: Simeprevir; SOF: Sofosbuvir; VEL: Velpatasvir; VOX: Voxilaprevir. * SOF + RBV represents an earlier-generation approach, but technicaly can be considered as pangenotypic.

**Table 3:** Comparison of patients with arterial hypertension who achieved sustained viral response to hepatitis C treatment with patients who did not achieve it.

Parameter	SVR Achieved (n = 5663)	SVR Not Achieved (n = 142)	*p*-Value
**HCV RNA (IU/mL), mean ± SD**	2.55 ± 0.79	2.34 ± 0.31	0.749
**Age, mean ± SD**	61.3 ± 12.1	59.7 ± 11.4	0.132
**Men/Women, % (n)**	45.6/54.4 (2581/3082)	58.4/41.6 (83/59)	0.002 (*χ*^2^ = 9.2)
**BMI, mean ± SD**	27.7 ± 4.8	28.1 ± 4.6	0.380
**Comorbidities**			
**Autoimmune diseases, % (n)**	2.2 (125)	2.8 (4)	0.627
**Malignancies other than HCC, % (n)**	2.7 (154)	3.5 (5)	0.563
**Diabetes, % (n)**	24.5 (1388)	22.5 (32)	0.589 (*χ*^2^ = 0.3)
**Renal disease, % (n)**	7.5 (424)	7.8 (11)	0.908 (*χ*^2^ = 0.1)
**CAD, % (n)**	8.5 (480)	9.9 (14)	0.56 (*χ*^2^ = 0.3)
**ALT >35 IU/I, % (n)**	79.3 (4489)	83.1 (118)	0.265 (*χ*^2^ = 1.2)
**Albumin < 3.5 g/dL, % (n)**	15.0 (848)	22.5 (32)	0.01 (*χ*^2^ = 6.2)
**Bilirubin > 1.2 mg/dL, % (n)**	12.9 (730)	28.2 (40)	<0.0001 (*χ*^2^ = 28.1)
**Hemoglobin (<12 g/dL women, <13 g/dL men), % (n)**	11.6 (655)	14.1 (20)	0.355 (*χ*^2^ = 0.9)
**Platelets <140 *x* 1000/µL, % (n)**	28.7 (1623)	52.8 (75)	<0.0001 (*χ*^2^ = 39.1)
**Creatinine >1.2 mg/dL, % (n)**	8.2 (463)	6.34 (9)	0.427
**Liver fibrosis**			
**F3, % (n)**	17.6 (994)	9.9 (14)	0.02 (*χ*^2^ = 5.7)
**F4, % (n)**	34.1 (1931)	59.9 (85)	<0.0001 (*χ*^2^ = 40.6)
**Child-Pugh Score B + C, % (n)**	3.4 (186)	12.7 (18)	<0.0001 (*χ*^2^ = 36.0)
**HCC history, % (n)**	2.2 (123)	6.3 (9)	0.001
**HCV genotype**			
**1a, % (n)**	1.7 (97)	0.7 (1)	0.382
**1b, % (n)**	81.8 (4632)	67.7 (96)	0.0002 (*χ*^2^ = 18.6)
**1, % (n)**	2.3 (132)	2.1 (3)	1.0
**2, % (n)**	0.1 (8)	0 (0)	-
**3, % (n)**	8.7 (495)	23.9 (34)	<0.0001 (*χ*^2^ = 38.7)
**4, % (n)**	3.6 (204)	2.8 (4)	0.819
**5, % (n)**	0.02 (1)	0.0 (0)	-
**6, % (n)**	0.04 (2)	0.0 (0)	-
**Unknown, % (n)**	1.6 (90)	2.8 (4)	0.293
**HBV coinfection, % (n)**	14.8 (820)	15.7 (22)	0.769 (*χ*^2^ = 0.9)
**HIV coinfection, % (n)**	2.0 (111)	2.9 (4)	0.465
**History of treatment failure, % (n)**	19.6 (1111)	27.5 (39)	0.02 (*χ*^2^ = 5.3)
**Current treatment**			
**ASV + DCV** **, % (n)**	1.0 (54)	4.2 (6)	0.003
**SOF/LDV ± RBV** **, % (n)**	19.3 (1093)	16.2 (23)	0.354 (*χ*^2^ = 0.9)
**OBV/PTV/r ± DSV ± RBV** **, % (n)**	22.0 (1248)	18.3 (26)	0.289 (*χ*^2^ = 1.1)
**GZR/EBR ± RBV** **, % (n)**	16.7 (945)	10.6 (15)	0.052 (*χ*^2^ = 3.8)
**SOF + SMV ± RBV,** **% (n)**	0.1 (3)	0	1.0
**SOF + RBV,** % (n) *	1.4 (81)	7.0 (10)	<0.0001 (*χ*^2^ = 28.3)
**SOF + DCV ± RBV**	0.2 (14)	0	1.0
**GLE/PIB** **, % (n)**	18.7 (1059)	14.8 (21)	0.237 (*χ*^2^ = 1.4)
**GLE/PIB + SOF + RBV** **, % (n)**	0.04 (2)	0	1.0
**SOF/VEL ± RBV** **, % (n)**	20.2 (1144)	28.2 (40)	0.012 (*χ*^2^ = 5.4)
**SOF/VEL/VOX, % (n)**	0.4 (20)	0.7 (1)	0.406
**Current-RBV-containing regimens, % (n)**	17.3 (977)	31.7 (45)	<0.0001 (*χ*^2^ = 19.9)

ALT: alanine transaminase; ASV: Asunaprevir; BMI: Body mass index; CAD: Coronary artery disease; DCV: Daclatasvir; DSV: Dasabuvir; EBR: Elbasvir; HBV: Hepatitis B virus; HCC: hepatocellular carcinoma; HCV: hepatitis C virus; HIV: Human immunodeficiency virus; GLE: Glecaprevir; GZR: Grazoprevir; LDV: Ledipasvir; OBV: Ombitasvir; r: Ritonavir; PTV: Paritaprevir; PIB: Pibrentasvir; RBV: Ribavirin; RNA: Ribonucleic acid; SMV: Simeprevir; SOF: Sofosbuvir; VEL: Velpatasvir; VOX: Voxilaprevir. * SOF + RBV represents an earlier-generation approach, but technicaly can be considered as pangenotypic.

**Table 4:** Safety of DAA therapy in hypertensive and non-hypertensive patients.

Parameter	Hypertensive Patients (n = 5976)	Non-Hypertensive Patients (n = 12,992)	*p*-Value
**Treatment course**			
**According to the schedule,** **% (n)**	96.5 (5769)	97.8 (12,707)	<0.0001 (*χ*^2^ = 35.1)
**Therapy modification,** **% (n)**	1.9 (111)	1.0 (128)	
**Therapy discontinuation,** **% (n)**	1.5 (89)	1.0 (126)	
**No data,** **% (n)**	0.1 (7)	0.2 (31)	
**Patients with at least one AE,** **% (n)**	21.4 (1280)	15.4 (1999)	<0.0001 (*χ*^2^ = 104.2)
**Serious adverse events,** **% (n)**	2.2 (129)	1.1 (141)	<0.0001 (*χ*^2^ = 33.6)
**AEs leading to treatment discontinuation,** **% (n)**	1.0 (57)	0.4 (58)	<0.0001 (*χ*^2^ = 17.5)
**Most common AEs (≥ 2%)**			
**Weakness/fatigue,** **% (n)**	8.4 (499)	6.1 (788)	<0.0001 (*χ*^2^ = 33.8)
**Anemia,** **% (n)**	2.1 (123)	0.9 (111)	<0.0001 (*χ*^2^ = 48.7)
**Headaches,** **% (n)**	2.3 (137)	2.5 (326)	0.369 (*χ*^2^ = 0.8)
**Itchy skin,** **% (n)**	2.3 (135)	1.3 (168)	<0.0001 (*χ*^2^ = 24.3)
**Hepatic AEs** **% (n)**			
**Ascites,** **% (n)**	1.9 (39)	3.0 (76)	0.014 (*χ*^2^ = 6.1)
**Hepatic encephalopathy,** **% (n)**	1.2 (25)	1.9 (47)	0.072 (*χ*^2^ = 3.2)
**Gastrointestinal bleeding,** **% (n)**	0.3 (7)	0.8 (19)	0.06 (*χ*^2^ = 3.5)
**Death,** **% (n)**	0.8 (47)	0.5 (68)	0.030 (*χ*^2^ = 4.7)

AEs: adverse effects, DAA: direct-acting antivirals.

## Data Availability

The data that support the findings of this study are available from the corresponding author upon reasonable request.
